# Parotid carcinoma following chronic lymphocytic inflammation with pontine perivascular enhancement responsive to steroids: a case report

**DOI:** 10.1186/s12883-021-02135-6

**Published:** 2021-03-10

**Authors:** Yilun Deng, Bi Zhao, Bing Wei, Shihong Zhang, Ming Liu

**Affiliations:** 1 Department of Neurology, West China Hospital, Sichuan University, Chengdu, Sichuan China; 2 Department of Pathology, West China Hospital, Sichuan University, Chengdu, Sichuan China

**Keywords:** CLIPPERS, PNS, Parotid carcinoma

## Abstract

**Background:**

Chronic lymphocytic inflammation with pontine perivascular enhancement responsive to steroids (CLIPPERS) is an inflammatory disorder with unclear causes. Paraneoplastic etiology may be a cause. We report a case of CLIPPERS with parotid carcinoma.

**Case presentation:**

A 54-year-old man with a history of lymphoma was hospitalized with a pontocerebellar syndrome. Brain MRI revealed that the pons and cerebellum were “peppered” with punctate and curvilinear enhancement lesions that supported the diagnosis of CLIPPERS. The relapse of lymphoma was excluded by a further cerebellum biopsy revealing predominantly CD3+ T cells in white matter. The patient was relieved after pulse therapy with intravenous methylprednisolone and a large dose of corticosteroids, but he complained of a worsening gait problem when corticosteroids were tapered to a lower dose. Although the clinical symptoms gradually improved again by increasing the dosage of corticosteroids with Azathioprine, the patient still had a slight unsteady gait during follow-up. At the 7-month follow-up, a parotid mass was detected by MRI and was verified as carcinoma by biopsy. After resection of parotid carcinoma, the residual symptoms and previous MRI lesions disappeared, and no relapse occurred.

**Conclusions:**

CLIPPERS may not be a distinct nosologic entity but an overlapping diagnosis with other diseases. Some cases of CLIPPERS might be a subtype of paraneoplastic neurological syndromes (PNS) due to the similar mechanism of antibody-mediated encephalitis. Tumor screening and serum paraneoplastic autoantibody tests are recommended for patients with CLIPPERS, especially for those who relapse when corticosteroids treatment is stopped or tapered.

**Supplementary Information:**

The online version contains supplementary material available at 10.1186/s12883-021-02135-6.

## Background

Chronic lymphocytic inflammation with pontine perivascular enhancement responsive to steroids (CLIPPERS) is a brainstem-predominant inflammatory syndrome with punctate and curvilinear enhancing lesions on brain MRI initially defined by Pittock [1]. These lesions are mainly distributed mainly in the pons and cerebellum and can extend, in a lesser degree, to the medulla, midbrain, brachium pontis and cortical-subcortical regions [2].

The pathogenesis of CLIPPERS remains unknown. The predominance of T-cells on histopathology and responsiveness to steroids treatment support an immune-mediated or other inflammatory causes; moreover, very few cases have reported on the role of paraneoplastic etiology during the disease cascade [2–4]. Here, we present the first case of CLIPPERS with parotid carcinoma, a combination that has not previously been reported in the literature.

## Case presentation

**Fig. 1:**
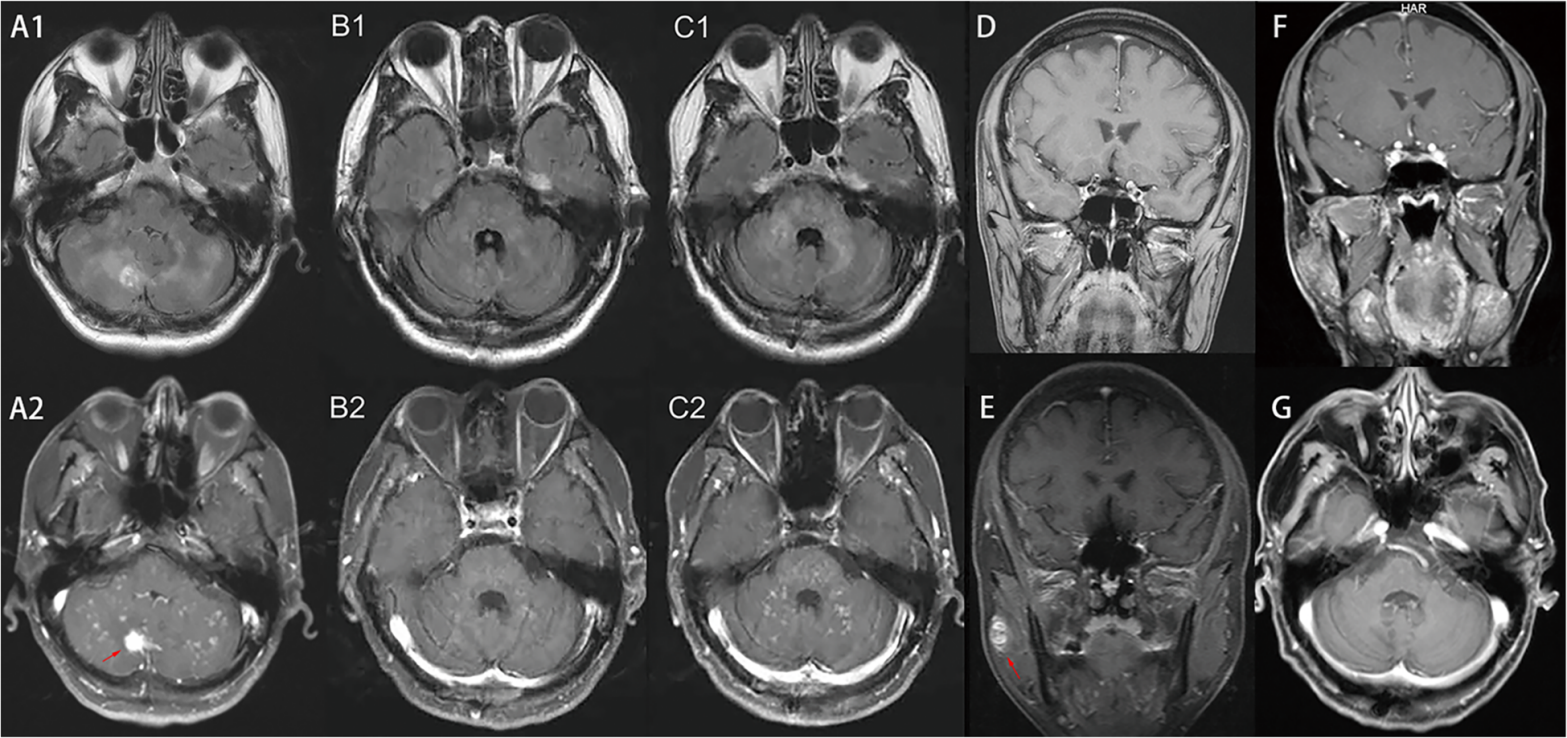
Radiological findings. Flair images (A1) and Axial contrast-enhanced T1-weighted (A2) showed punctate and curvilinear enhancing lesions “peppering” on pons and cerebellum, with a large one in 1.07 cm diameter located in the right cerebellar hemisphere (arrow). There was no parotid malignancy observed at that time (D). With pulse therapy of intravenous methylprednisolone and following oral prednisolone, subsequent MRI showed the lesions decreased both in size and amount (B1, B2). When the dosage of prednisolone was tapered to 20 mg two months later, MRI showed recurring lesions (C1, C2). An enhancing nodule of 1.8 cm diameter was found in right masseter muscle (arrow) (E). After the resection of parotid malignancy (F), with adjuvant radiotherapy, PCGE lesions disappeared on MRI (G)

In July 2017, a 54-year-old Chinese male with progressive gait disturbance, dysarthria and intermittent diplopia for more than 2 months was admitted. He had a history of cured Hodgkin’s lymphoma 9 years prior. The neurological examination revealed horizontal nystagmus, mild dysarthria, ataxia gait and impaired coordinated movements of limbs. MRI T2-weighted and fluid attenuated inversion recovery sequence images revealed multiple patchy hyperintense lesions bilaterally scattered in the pons, cerebellum, midbrain and middle cerebellar peduncle with nodular gadolinium enhancing (Fig. 1. A1, A2). Positron emission tomography-CT had been performed in clinic before admission revealed abnormal accumulation of fluorodeoxyglucose in the right cerebellum, which was concerning for a recurred lymphoma. In addition, lumbar puncture was performed in another hospital before admission which only demonstrated a mildly elevated protein concentration (84 mg/dl) and an unremarkable cytology. Cerebellar biopsy was performed and it demonstrated parenchymal and perivascular lymphocytic inflammatory infiltrate in the white matter (Supplementary information. Figure S1. A). Serum serological tests for syphilis and human immunodeficiency virus were negative. Except for a mildly elevated antinuclear antibodies titer (1:100), the other autoimmune antibody tests were negative. Cerebrospinal fluid (CSF) examination revealed a mildly elevated protein concentration (77 mg/dl). Cytology, flow cytometry, immunoglobulin G index and culture of the CSF were unremarkable. Given the lack of evidence for lymphoma, a possible autoinflammatory etiology was considered. A 5-day course of intravenous immunoglobulin (400 mg/day per kilo of weight) was given and the patient was discharged.

One month later, the patient was readmitted with no clinical and radiological improvement. This time, CLIPPERS was considered. Pulse therapy with methylprednisolone at 1 g per day was given for 5 consecutive days, followed by 60 mg of oral prednisolone therapy (1 mg/kg body weight) per day, slowly tapering to 20 mg. The clinical symptoms improved significantly, and MRI showed a decrease in the number and size of punctate and curvilinear gadolinium-enhancing (PCGE) lesions in the cerebellum and pons (Fig. 1. B1, B2). Two months later, when the daily dosage of oral prednisolone was reduced to 20 mg, the patient complained of recurring gait disturbance. Brain MRI revealed an increased number of PCGE lesions (Fig. 1. C1, C2). The clinical symptoms gradually resolved when the daily dose of oral prednisolone was increased to 50 mg. Azathioprine was added, and there was no recurrence, but the patient had a slight unsteady gait during follow-up.

**Fig. 2:**
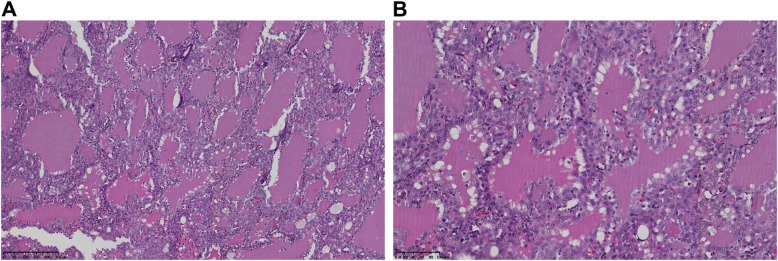
The pathology of the right parotid biopsy. Tumor forms follicles, which contain eosinophilic secretory material, resemble to thyroid follicles (haematoxylin and eosin stain). (**a**; × 100, **b**; × 200)

Seven months later, MRI detected an enhancing nodule in the right masseter muscle (Fig. 1. E). The biopsy demonstrated parotid carcinoma (Fig. 2). After resection of the parotid carcinoma and subsequent radiotherapy, with daily dosages of 10 mg oral prednisolone and 50 mg azathioprine, the residual symptoms associated with CLIPPERS disappeared. To date, the patient is free of recrudescence of CLIPPERS, and the PCGE lesions disappeared on follow-up MRI (Fig. 1. G). A serum paraneoplastic autoantibody panel including anti-Hu, anti-Yo, anti-Ri, anti-CV2, anti-Ma1, anti-amphiphysin and anti-Tr was negative.

## Discussion and conclusions

CLIPPERS is a chronic lymphocyte inflammation disorder predominantly infiltrating the pons and cerebellum. Our patient’s clinical symptoms, histopathological characteristics, responses to prednisolone therapy, and MRI findings except for a large mass (> 3 mm in diameter) in cerebellum were consistent with the diagnostic criteria of CLIPPERS proposed by Tobin et al. [5]. Previously the diagnostic criterion of CLIPPERS according to brain MRI features, gadolinium enhancing nodules scattered in the pons and cerebellum were less than 3 mm in diameter; however, Taieb and his colleagues further evaluated the diagnostic criterion and suggested that nodular enhancement (> 3 mm in diameter) can be included in the diagnostic criteria because the previous criterion lacked sensitivity and specificity [6]. During prolonged follow-up, the authors found that patients who initially didn’t meet the diagnostic criteria of CLIPPERS due to nodular gadolinium enhancing (> 3 mm), later met the diagnostic criteria of definite CLIPPERS and probable CLIPPERS because of their clinical course, neuroimaging appearance and pathological findings [6]. In the reply letter by Tobin et al. to Taieb and coworkers [7], he agreed with the suggestion that enhancement lesions > 3 mm should not completely be ruled out, and required a further brain biopsy in the absence of alternative diagnosis in noninvasive investigations.

CLIPPERS is mediated by an autoimmune process without specific antibodies, which suggests it might not be a distinct nosological entity but a diagnosis that overlaps with other diseases. Paraneoplastic neurological syndromes (PNS) are inflammatory disorder predominantly mediated by T cells, and immune therapy lacks efficacy which is similar with CLIPPERS when steroids are weaned [8]. Specific antibodies have not been detected in CLIPPERS and many onconeural antibodies of PNS are unknot identified as well [8]. Many cases initially presented with clinical and radiological features of CLIPPERS, and then lymphoma is diagnosed in follow-up [9, 10] . In addition to lymphoma, histiocytic sarcoma with an initial manifestation of CLIPPERS was reported as well [11]. CLIPPERS, a pre-stage of malignancy, may represent a compensatory and temporary host immune response, however, when the response becomes too weak, the lymphoma, histiocytic sarcoma might emerge [11, 12]. In addition, a similar mechanism might be applied to other malignancies. To the best of our knowledge, the patient we reported was the first case of CLIPPERS with an associated parotid carcinoma. Of note, after removal of parotid carcinoma, clinical symptoms and the PCGE lesions on MRI disappeared without relapse during long-term follow-up. Although common PNS antibodies were not found in our patient, this might be due to the fact that the testing was performed after the therapy of carcinoma and corticosteroid treatment, or a specific antibody associated with CLIPPERS in parotid carcinoma has not been identified.

There is a reported case of CLIPPERS where whole body gallium scan showed abnormal uptake in the parotid, and biopsy revealed chronic lymphocytic sialadenitis [13] . Noteworthily, a benign lesion in parotid might progress into malignancy. A reported case with a fullness at the angle of the right jaw, and the histopathological findings indicated sialadenitis. Two years later the patient complained of sudden painful enlargement of her right face, and a second biopsy demonstrated parotid acinic cell carcinoma with acute parotitis [14]. Therefore, long term follow-up might be needed to detect the progression of a benign lesion in the parotid even though the risk of missing a malignancy is small.

Whether CLIPPERS is an inflammatory disease or an overlapping syndrome with other diseases remains unknown. With an increasing number of cases reported in recent years, it is conceivable that CLIPPERS might be a pre-stage or unique presentation of other well-determined diseases, such as lymphoma [10, 15], hemophagocytic syndrome [11], neuromyelitis optica spectrum disorders or other diseases [16]. Noteworthily, in the cases aforementioned, the diagnosis of CLIPPERS took a long duration (several months to 10 years), therein, diagnosis of CLIPPERS needs a long period of observation and monitoring. When patients are less responsive to corticosteroid therapy, it is suggested to re-evaluate the CLIPPERS criteria even though definite CLIPPERS was considered initially because it might suggest that the disease is in development and the underlying cause might be detected [6].

We would like to acknowledge some limitation in our report. This case is consistent with CLIPPERS according to the patient’s clinical manifestation, radiological features and histopathological characteristics, but staining of antibodies such as glial fibrillar acidic protein astrocytopathy may help make a thorough differential of other diseases.

In conclusion, our case demonstrated that CLIPPERS might be a premalignant state, and systemic tumor screening is recommended, especially of the parotid gland. In addition, long-term follow-up is required in cases where some tumors are difficult to detect at the early stage.

## Supplementary Information

**Additional file 1: Figure S1.** **The pathology of cerebellum biopsy**. The Nanozoomer 2.0 HT series (Hamamatsu, Japan) was used, which is a system that converts glass slides into digital slides using the time delay integration (TDI) line scanning. A single slide was scanned at a resolution of 1.9 billion pixels in 1 min 40 s. The measured resolution at which an image was acquired is 300 dpi. Neuropathology showed perivascular and parenchyma infiltration (haematoxylin and eosin stain) (A; × 100). CD3-positive T lymphocytes infiltrates predominantly (B × 100) with some CD20-positive B lymphocytes (C; × 100). CD4-positive T cells (D; × 400). The CD8-positive cells are less than 50% of CD3-positive cells (E; × 400). CD20-positive B cells (F; × 400).

## Data Availability

All data generated or analysed during this study are included in this published article [and its supplementary information files].

## Abbreviations

CLIPPERS: Chronic lymphocytic inflammation with pontine perivascular enhancement responsive to steroids
PNS: Paraneoplastic neurological syndromes
CSF: Cerebrospinal fluid
PCGE: Punctate and curvilinear gadolinium-enhancing
