# Supplementary figures and images for: Parotid carcinoma following chronic lymphocytic inflammation with pontine perivascular enhancement responsive to steroids: a case report
Source: BMC Neurol. 2021 Mar 10;21:110. doi: 10.1186/s12883-021-02135-6 (PMC7944626; doi:10.1186/s12883-021-02135-6)

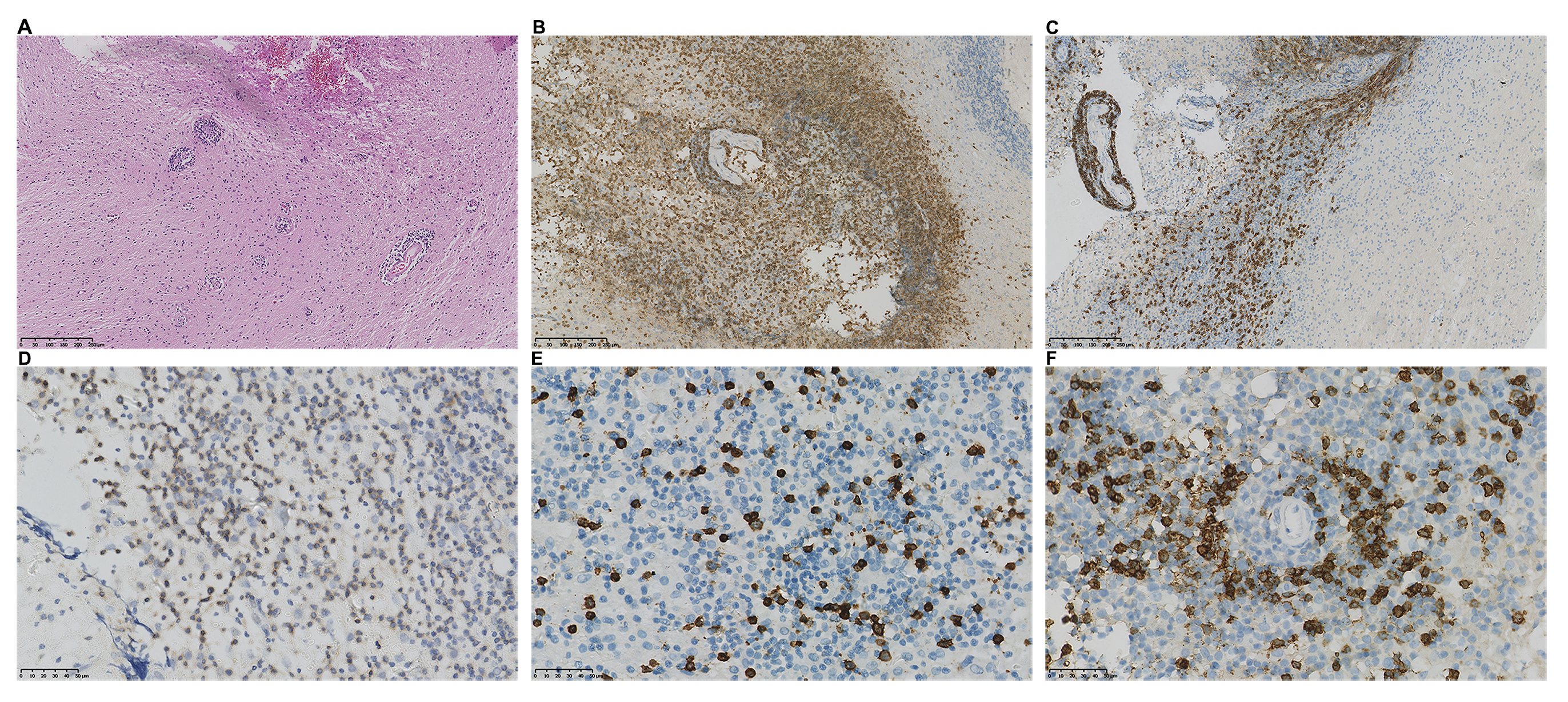

Supplement: Supplementary file 1 — Additional file 1: Figure S1. The pathology of cerebellum biopsy. The Nanozoomer 2.0 HT series (Hamamatsu, Japan) was used, which is a system that converts glass slides into digital slides using the time delay integration (TDI) line scanning. A single slide was scanned at a resolution of 1.9 billion pixels in 1 min 40 s. The measured resolution at which an image was acquired is 300 dpi. Neuropathology showed perivascular and parenchyma infiltration (haematoxylin and eosin stain) (A; × 100). CD3-positive T lymphocytes infiltrates predominantly (B × 100) with some CD20-positive B lymphocytes (C; × 100). CD4-positive T cells (D; × 400). The CD8-positive cells are less than 50% of CD3-positive cells (E; × 400). CD20-positive B cells (F; × 400). [file 12883_2021_2135_MOESM1_ESM.tif]
